# A rapid field test for the measurement of bovine serum immunoglobulin G using attenuated total reflectance infrared spectroscopy

**DOI:** 10.1186/s12917-015-0539-x

**Published:** 2015-08-20

**Authors:** Ibrahim Elsohaby, Siyuan Hou, J. Trenton McClure, Christopher B. Riley, R. Anthony Shaw, Gregory P. Keefe

**Affiliations:** Department of Health Management, Atlantic Veterinary College, University of Prince Edward Island, 550 University Avenue, Charlottetown, PEI C1A 4P3 Canada; Infectious Diseases, Department of Animal Medicine, Faculty of Veterinary Medicine, Zagazig University, Zagazig, 44511 Sharkia Province Egypt; Institute of Veterinary, Animal and Biomedical Sciences, Massey University, Palmerston North, 4442 New Zealand; National Research Council of Canada, Medical Devices Portfolio, Winnipeg, MB R3B 1Y6 Canada

**Keywords:** Bovine, Failure of transfer of passive immunity, Attenuated total reflectance infrared spectroscopy, Immunoglobulin G

## Abstract

**Background:**

Following the recent development of a new approach to quantitative analysis of IgG concentrations in bovine serum using transmission infrared spectroscopy, the potential to measure IgG levels using technology and a device better designed for field use was investigated. A method using attenuated total reflectance infrared (ATR) spectroscopy in combination with partial least squares (PLS) regression was developed to measure bovine serum IgG concentrations. ATR spectroscopy has a distinct ease-of-use advantage that may open the door to routine point-of-care testing. Serum samples were collected from calves and adult cows, tested by a reference RID method, and ATR spectra acquired. The spectra were linked to the RID-IgG concentrations and then randomly split into two sets: calibration and prediction. The calibration set was used to build a calibration model, while the prediction set was used to assess the predictive performance and accuracy of the final model. The procedure was repeated for various spectral data preprocessing approaches.

**Results:**

For the prediction set, the Pearson’s and concordance correlation coefficients between the IgG measured by RID and predicted by ATR spectroscopy were both 0.93. The Bland Altman plot revealed no obvious systematic bias between the two methods. ATR spectroscopy showed a sensitivity for detection of failure of transfer of passive immunity (FTPI) of 88 %, specificity of 100 % and accuracy of 94 % (with IgG <1000 mg/dL as the FTPI cut-off value).

**Conclusion:**

ATR spectroscopy in combination with multivariate data analysis shows potential as an alternative approach for rapid quantification of IgG concentrations in bovine serum and the diagnosis of FTPI in calves.

## Background

Immunoglobulins are glycoproteins produced by B-lymphocytes, and are a crucial component of the host’s adaptive immune system [1]. Immunoglobulin G (IgG) is the predominant class of immunoglobulins involved in transfer of passive immunity to newborn calves via colostrum [2]. Failure of transfer of passive immunity (FTPI) occurs when calves fail to ingest or absorb sufficient IgG (<1000 mg/dL) from colostrum [3]. FTPI is a major predisposing risk factor for early neonatal losses associated with gastroenteritis, pneumonia, and septicemia [3, 4]. Reduced long-term productivity, decreased milk yield, and increased culling rates during first lactation have also been associated with FTPI [5, 6]. The monitoring of IgG levels is important for assessment of farm colostrum management and to reduce productivity losses associated with calf-hood diseases [7].

A number of methods have been used to measure the IgG concentrations in bovine serum. The most widely accepted reference method used is the radial immunodiffusion (RID) assay that provides direct measurement of IgG concentrations in bovine serum [8]. However, RID has significant drawbacks, including the time it takes to obtain results (18–24 h), utilization of labile reagents, high cost [9, 10], and discrepancies among RID kits due to inaccuracies associated with the internal standards [11]. Other methods, such as refractometry, zinc sulfite turbidity test, sodium sulfate turbidity test, serum γ-glutamyl transferase activity, whole blood glutaraldehyde coagulation test, and ELISA have been used to identify calves with FTPI with varying degrees of accuracy [2, 12, 13]. Despite these options, there is a need for an accurate, simple, rapid, and cost-effective method for quantification of IgG concentrations in bovine serum and for diagnosis of FTPI in dairy calves, which may be readily translated to the farm, clinic or small laboratory setting.

Infrared (IR) spectroscopy is one of the most important tools in modern analytic chemistry that is used for quantitative and qualitative analyses of many sample types [14, 15]. To obtain IR spectra from these samples various analyzing techniques have been used including transmission and attenuated total reflectance methods. For transmission techniques, the sample is directly placed into the path of the IR beam, and then the transmitted energy is measured and a spectrum is generated [16, 17]. However, the ATR technique differs in that it measures changes that occur to the totally internally reflected IR beam after the beam comes into contact with a sample [16, 17].

Transmission IR spectroscopy, in combination with partial least squares (PLS) regression, has been used to measure total serum protein, glucose, albumin, triglyceride, cholesterol, and urea in human serum samples [14, 18, 19]. In veterinary applications, it is also widely used for nutrient analysis of milk [20, 21], and screening of dairy cows for metabolic diseases such as ketosis [22]. Technical difficulties commonly encountered with transmission techniques can make routine collection of high-quality spectra in the field situation difficult [23]. These difficulties include practical issues associated with filling and cleaning short-pathlength cells (for spectroscopy of liquid samples), uncertainties in optical pathlength (for spectroscopy of dried films), and the time required for sample submission and preparation [16, 17]. In contrast to transmission IR spectroscopy, attenuated total reflectance infrared (ATR) spectroscopy by its nature does not have issues associated with optical pathlength or sample thickness. The measurement is simple and fast, and requires little or no sample preparation [24]. For these reasons, ATR has emerged as one of the most commonly used IR spectroscopic analyzing techniques, with applications addressing various clinical diagnostic problems in human and veterinary medicine [25–27]. Most recently, robust, small footprint ATR equipment has been manufactured that is ideally suited for field use on the farm, veterinary clinic or small laboratory [28].

The objectives of this study were to investigate the potential use of ATR, in combination with multivariate data analysis, for the rapid quantification of IgG concentrations in bovine serum, and for the diagnosis of FTPI in dairy calves. This study also investigated the effects of different spectral data pre-processing techniques on model performance and predictive accuracy.

## Methods

### Serum samples

Serum samples (*n* = 250) from Holstein-Friesian calves and adult dairy cows were used. Calf samples (*n* = 208, including eight prior to colostrum ingestion) had previously been collected for companion studies [29, 30], and adult dairy cow samples (*n* = 42) had been collected for the Maritime Quality Milk laboratory (Charlottetown, Prince Edward Island, Canada). Samples were stored at -80 °C at the University of Prince Edward Island before use. All samples were tested at the same time, using a radial immunodiffusion (RID) assay to quantify the serum IgG level and ATR spectroscopy to acquire the IR spectrum for each sample. The research protocol was reviewed and approved by the Animal Care Committee at University of Prince Edward Island (Protocol number: 6006206).

### RID assay for IgG

A commercial RID assay (Bovine IgG RID Kit, Triple J Farms; Bellingham, WA) was used as the reference method for determining serum IgG concentrations. Serum samples were thawed at room temperature and then vortexed for 10 s. Subsequently, IgG was measured using the bovine RID assay with a working range of 196 to 2748 mg/dL. For the eight samples that were collected prior to colostrum ingestion, IgG was quantified using the Bovine Ultra Low Level IgG RID Kit with a working range of 10–100 mg/dL. Each RID assay was performed according to the manufacturer’s instructions using 5 μL of undiluted serum in each well. The diameters of the precipitating zones were measured after 18–24 h by the same individual, using a handheld caliper. Each sample and manufacturer’s standards were tested in replicates of five, with one replicate per RID plate, and the mean values were calculated. The assay standards were used to construct a calibration curve that was used to determine the IgG concentrations of the study serum samples.

### ATR assay for IgG

Infrared spectra were acquired using a customized 3-bounce attenuated total reflectance mid-infrared spectrometer (Cary 630 IR spectrometer, 3B Diamond ATR Module ZnSe element, Agilent Technologies, Dansbury, Connecticut). Thawed serum samples were diluted (1:1) with deionized sterile water and vortexed at a maximum of 2700 rpm for 10 s to homogenize the samples. Following dilution, 5 μL aliquots were evenly spread onto the ATR element of the optics module of the spectrometer and dried by a stream of air from a domestic hair dryer. The sample was completely dried within 3-4 min and formed a thin film on the optical element.

Spectra were collected over the wavenumber range of 4000–650 cm^−1^ with a nominal resolution of 8 cm^−1^. For each spectrum, 32 scans were co-added to increase the signal-to-noise ratio. Before each measurement, the stage of the optics module of the spectrometer was cleaned with 100 % ethanol and allowed to dry, and a new background reading was collected. Each serum sample was tested in replicates of five. A total of 1250 (250 × 5) ATR spectra were collected and saved in GRAMS spectrum (SPC) format (GRAMS/AI version 7.02, Thermo Fisher Scientific), and then converted into PRN (printable) formatted data. The PRN format spectral data were imported into MATLAB® (version R2012b, MathWorks, Natick, MA) and further data analysis was performed using scripts written by the authors.

### Spectral pre-processing

Several pre-processing methods, including Savitsky-Golay smoothing (2^nd^ order polynomial function with 9 points), first-order and second-order derivative spectra [31], and two different normalization methods (Standard normal variate (SNV) and vector normalization) [32, 33] were applied to examine effects, if any, on the calibration models. This was followed by spectrum region selection of the 3700–2600 cm^−1^ and 1800–1300 cm^−1^ wavenumber regions, which exhibited the strongest absorptions in the original spectra. With five replicate spectra per serum sample, spectrum outlier detection was performed using Dixons Q-test [34, 35] at each wavenumber. If absorbance values were outliers (95 % confidence level) for over 50 % of the spectral data points for a given spectrum, that complete spectrum was treated as an outlier and excluded from further analysis. The average of the replicate spectra for each sample (after removal of outliers) was used for subsequent analysis.

### Multivariate calibration model development

The 250 serum samples were sorted based on IgG concentrations obtained from the RID assays. Serum samples with IgG concentrations outside of the manufacturer’s stated performance range for the RID assay were excluded from further analysis (*n* = 50; 28 calves; 22 cows). The remaining samples (*n* = 200) were linked to their corresponding pre-processed IR spectra, and then split into two sets (prediction and calibration sets). The prediction set (*n* = 67) was identified by ordering all of the serum samples according to their corresponding IgG levels and selecting the spectra of every third serum sample as a member of the prediction set. Thus, the prediction set encompassed the full range of IgG values for use in testing the predictive performance of the calibration models. The remaining calibration set (*n* = 133) was randomly split into training (*n* = 67) and validation (*n* = 66) data sets for model development.

Calibration models were developed using PLS regression to relate spectroscopic features (pre-processed wavenumber bands) to the reference serum RID IgG concentrations. PLS regression was applied first to the training set, with up to 30 PLS factors retained, to develop 30 trial calibration models. Each of these models was employed to calculate the IgG concentration of each sample in the validation set, and an error estimate (sum of the squares of the differences between RID IgG values and the ATR predicted IgG values) was calculated. This procedure was repeated 10,000 times (including new randomly assigned splits of the calibration data set into new training and validation sets). The root mean squared error for the Monte Carlo cross validation value (RMMCCV) [36, 37] was calculated for each of the 30 trial calibration models, and then the optimal number of PLS factors was chosen as the one giving the lowest RMMCCV. Once the number of PLS factors was determined, the training and validation sets were combined (i.e., all the samples in the calibration set) to evaluate a PLS calibration model with the determined number of PLS factors.

### Evaluation of the calibration models

The predictive accuracy of each multivariate calibration model was assessed using the independent prediction data set (*n* = 67). The level of agreement between IgG concentrations measured by RID assay and predicted by ATR assay was first assessed for both the calibration and prediction sets by a scatter plot, Pearson correlation coefficient, and the concordance correlation coefficient. This was followed by a Bland–Altman plot [38, 39], which was used to examine the differences between RID and ATR assays values for the prediction set, thereby assessing the interchangeability of the two assays.

The precision of both the ATR and RID methods was investigated using the prediction set samples. The mean and standard deviations (SD) were calculated for the five replicates of each serum sample, and from these a modified coefficient of variation (CV* = CV (1 + (1/4n)); *n* = 5) was determined [40]. For each sample, and for each analytical method, the CV* was then plotted against the mean IgG concentration.

Finally, the potential utility of the ATR method was further assessed using the ratio of predictive deviation (RPD: the population standard deviation of the RID-determined IgG concentrations, ratioed to the RMSEP for the ATR method), and the range error ratio (RER: the range of the RID-determined IgG concentrations, ratioed to the RMSEP for the ATR method) [41]. According to this framework, a RPD <2 is considered to be poorly predictive; values between 2.0 and 2.5 are adequate for qualitative evaluation or for screening purposes; values >2.5 (or RER >10) are regarded as acceptable for quantification; and values >3 (or RER >20) suggest that the model is suitable for very accurate quantitative analysis [41].

### Clinical utility of ATR assay

To evaluate the clinical utility of the ATR assay for the diagnosis of FTPI (serum IgG <1000 mg/dL) in dairy calves, the test characteristics were calculated using 2×2 tables in prediction and entire data sets. These calculations were performed with Stata version 13.0 statistical software (StataCorp, College Station, TX). Sensitivity (Se) was defined as the proportion of samples with FTPI, as determined by RID, that were classified as positive by the ATR assay. Conversely, specificity (Sp) was defined as the proportion of samples without FTPI that were classified as negative by the ATR assay. Accuracy was defined as the proportion of samples that were correctly classified by the ATR assay.

## Results

### RID assay

The IgG concentrations of the serum samples with concentrations within the range of the RID assays (*n* = 200) ranged from 5.5 to 2983 mg/dL, with an average and SD of 1122 and 866 mg/dL, respectively. Separate IgG concentration statistics (mean, SD and range) for the calibration set and the prediction set are summarized in Table 1.

**Table 1:** Descriptive statistic of immunoglobulin G (IgG) concentrations of 200 bovine serum samples, measured by the reference method of radial immunodiffusion (RID) assay in the calibration and prediction data sets

Item	Calibration set				Prediction set			
	N	Mean	SD	Range	N	Mean	SD	Range
RID IgG	133	1117	864	2975	67	1132	876	2977

*N* Number of samples in the calibration and prediction sets, *SD* standard deviation (mg/dL), *range* Difference (highest minus lowest) of IgG concentration (mg/dL)

### ATR spectra

**Fig. 1:**
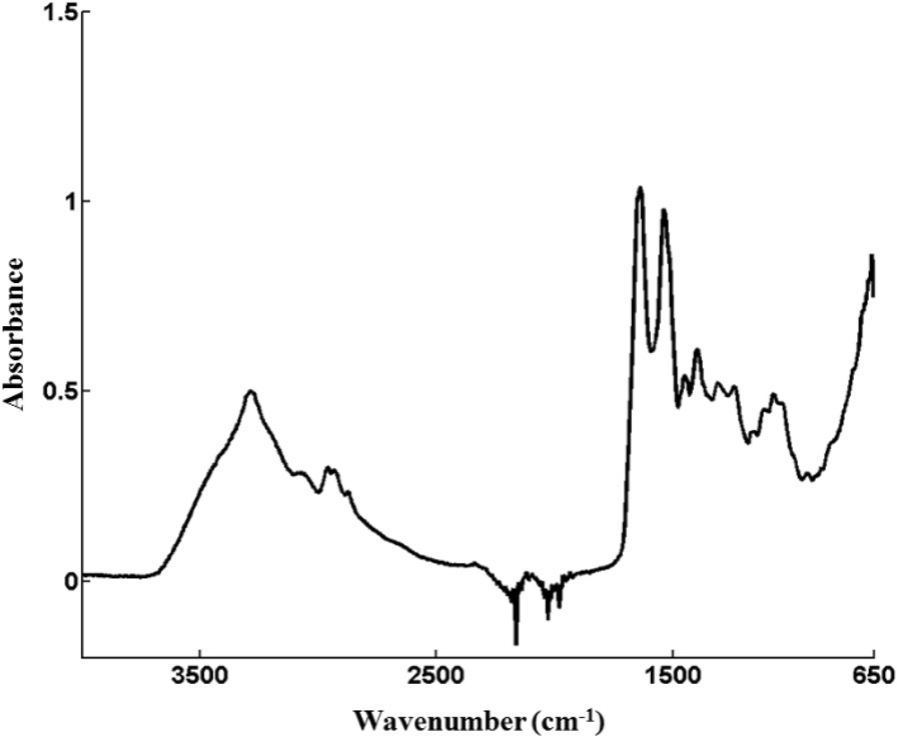
A representive raw spectrum of a bovine serum sample over the spectral range of 4000 – 650 cm^−1^ obtained by attunuated total reflectance infrared spectroscopy

A typical ATR spectrum of bovine serum over wavenumber range of 4000–650 cm^−1^ is shown in Fig. 1. Strong absorption bands at 1650 cm^−1^ and 1550 cm^−1^, correspond to C = O stretching and N–H bending vibrations respectively, while a broad strong absorption band centered at 3300 cm^−1^ was attributed to N–H stretching vibration [14, 15]. The noise in the 2200–1900 cm^−1^ range was due to absorptions by the diamond coating on the ZnSe ATR element. The spectral region at 4000–3800 cm^−1^ was a true baseline, free of appreciable sample absorptions, and as such was highly reproducible for all spectra.

### Multivariate calibration model

**Fig. 2:**
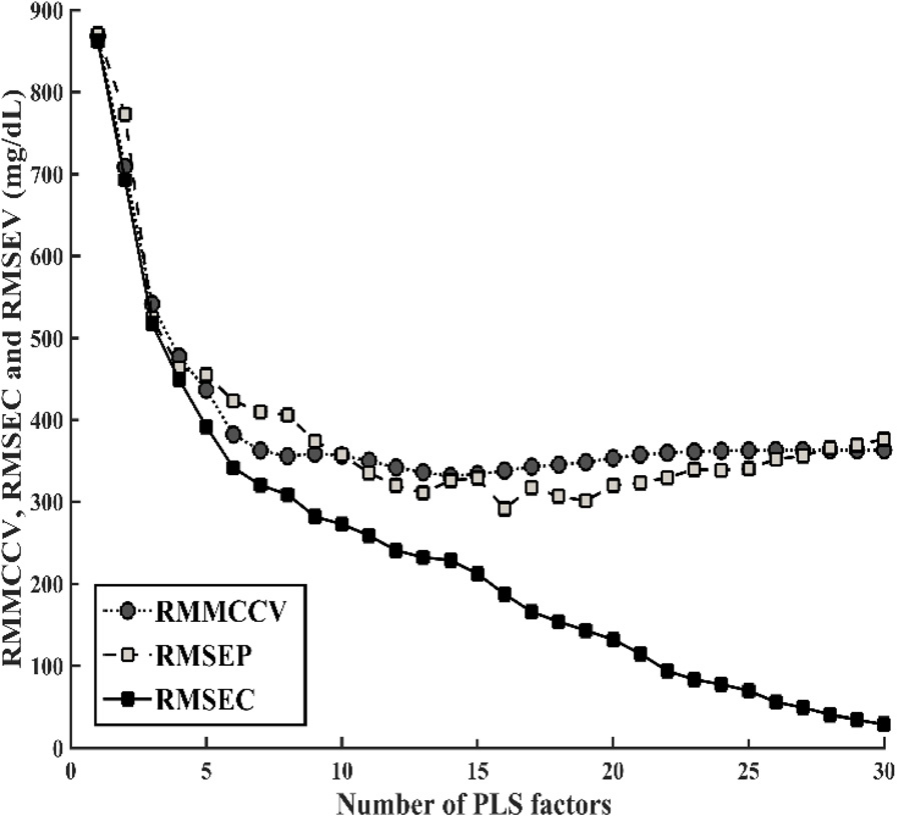
Plots of RMMCCV and RMSEC for the calibration (*n* = 133) data set, and RMSEP for the prediction (*n* = 67) data set. The optimum number of PLS factors was determined to be 14, based on the lowest RMMCCV. RMMCCV: Root mean squared error in the Monte Carlo cross validation value; RMSEC: Root mean squared error of calibration; RMSEP: Root mean squared error of prediction; PLS: Partial least squares

Trial PLS models were built to compare several pre-processing methods. The optimized results were obtained using data from the smoothed spectra with a 9 point Savitzky-Golay filter and SNV scaling (Table 2). The optimum number of PLS factors for this model was 14 (Fig. 2), based on the lowest IgG RMMCCV (332 mg/dL). Fig. 2 also shows root mean squared error of calibration (RMSEC) and root mean squared error of prediction (RMSEP) plotted against the number of PLS factors.

**Table 2:** Comparison of calibration models and prediction results of the immunoglobulin G (IgG) concentration of 200 bovine serum samples, obtained using different pre-processing approaches for infrared spectra

			Calibration (*n* = 133)		Prediction (*n* = 67)			
Pre-processing	PLS factors	RMMCCV (mg/dL)	*r*	RMSEC (mg/dL)	*r*	RMSEP (mg/dL)	RPD	RER
Smoothing (9 points)	14	335	0.97	364	0.92	340	2.6	8.8
Smoothing + normalization (SNV)	14	332	0.97	359	0.93	326	2.7	9.1
Smoothing + vector normalization	14	336	.097	367	0.93	331	2.7	9
1^st^ derivatives (9 points)	6	341	0.95	370	0.91	373	2.4	8
1^st^ derivatives + normalization (SNV)	6	338	0.95	355	0.91	362	2.4	8.2
1^st^ derivatives + vector normalization	6	340	0.96	357	0.91	362	2.4	8.2
2^nd^ derivatives (9 points)	5	363	0.97	382	0.88	424	2.1	7
2^nd^ derivatives + normalization (SNV)	5	358	0.97	376	0.87	429	2	7
2^nd^ derivatives + Vector normalization	6	356	0.97	376	0.89	409	2.1	7.3

*PLS*, Partial least squares, *RMMCCV* Root mean squared error of the Monte Carlo cross validation value, *r* Pearson correlation coefficient, *RMSEC* Root mean squared error of calibration;, *RMSEP* Root mean squared error of prediction, *RPD* (ratio of predictive deviation), *SD* divided by RMSEP, *RER* (range error ratio), *Range* divided by RMSEP; *SNV* Standard normal variate

### Calibration model validation

**Fig. 3:**
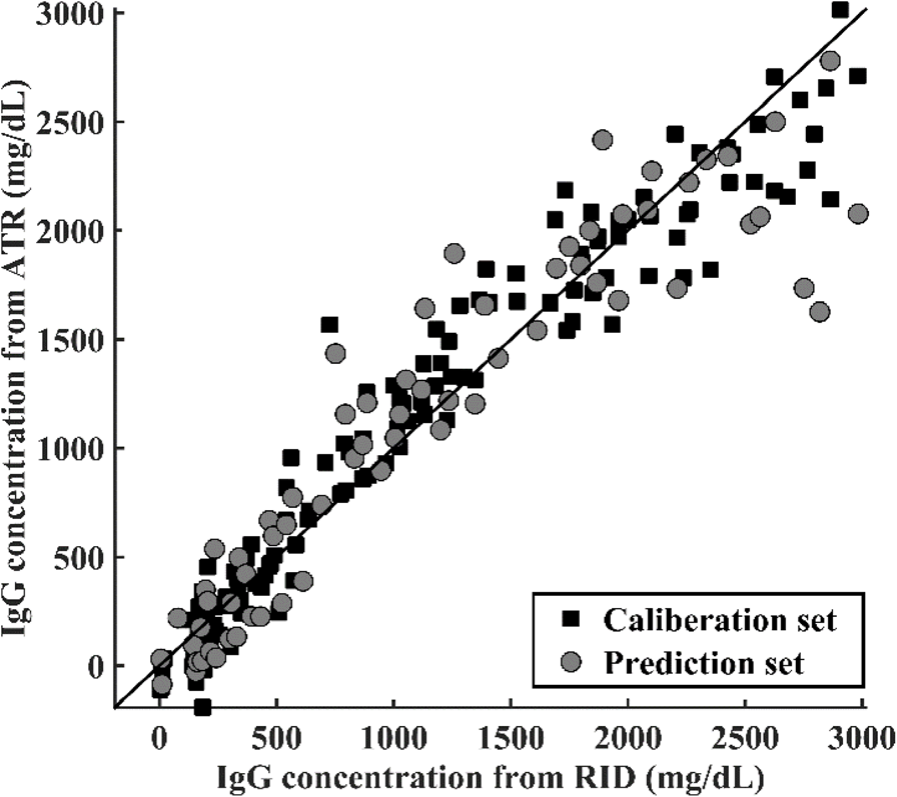
Scatter plots comparing immunoglobulin G (IgG) concentrations measured by the radial immunodiffusion (RID) assay to those predicated by the ATR-based assay for 200 bovine serum samples. The correlation coefficients (*r*) were 0.97 and 0.93 for the calibration and prediction data sets, respectively. The squares denote the samples from the calibration set and the circles indicate the samples from the prediction set. The two assays are considered comparable if data points distribute closely about the reference line

Figure 3 shows a scatter plot that indicates the level of agreement between IgG concentrations measured by the RID assay and those predicted by ATR spectroscopy for the calibration and prediction sets. The plots for the prediction set showed dispersions similar to those for the calibration set, with no significant over-fitting or under-fitting observed, indicating that a robust calibration model was developed. The Pearson correlation coefficients (*r*) for the calibration and prediction sets were 0.97 and 0.93, respectively. The concordance correlation for the calibration set was 0.97 and for the prediction set 0.93.

**Fig. 4:**
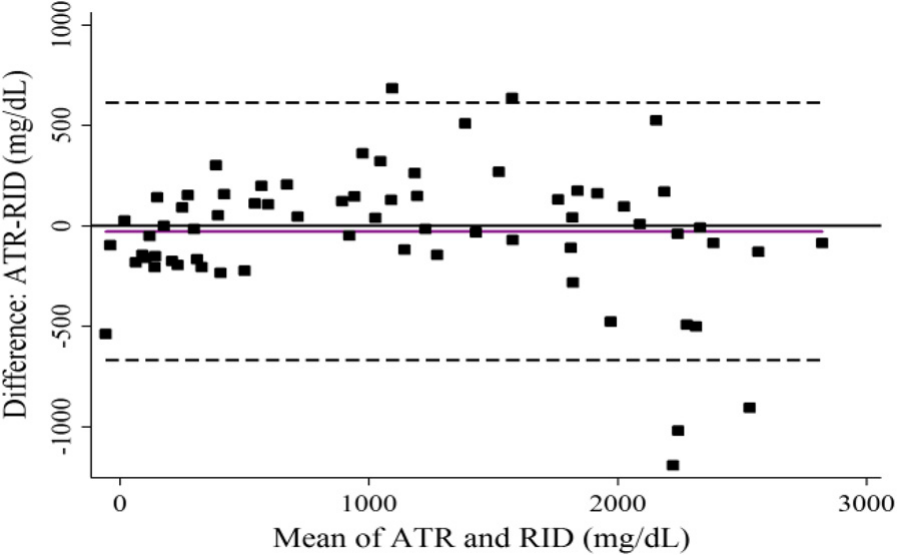
Bland–Altman plot. The average immunoglobulin G (IgG) concentrations measured by radial immunodiffusion (RID) and ATR methods (x-axis) against the difference in IgG concentrations between the two methods (y-axis) for the prediction set samples (*n* = 67). The dashed lines represent the 95 % confidence limits of agreement (–670 to 611 mg/dL) and the solid line represents the mean difference between ATR and RID assays (-30 mg/dL), indicating no appreciable systematic difference between the two methods

**Fig. 5:**
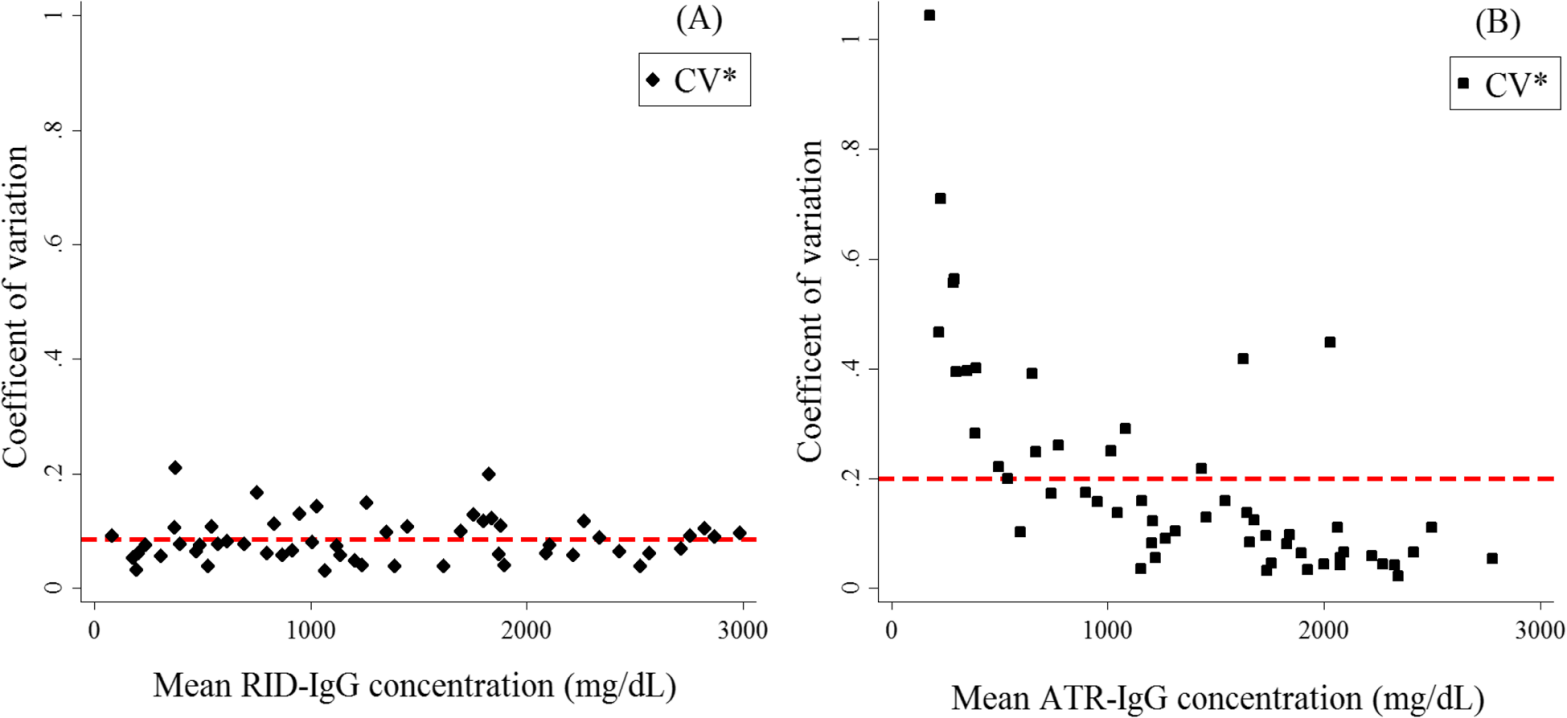
Adjusted coefficient of variance (CV*) plots for (**a**) the radial immunodiffusion (RID) method and (**b**) the ATR-based method for the prediction (*n* = 67) data set. The dashed line represents the mean CV* for RID assay (0.086, or 8.6 %) and ATR (0.2, or 20 %)

The Bland-Altman plot (Fig. 4) revealed that the mean value of the differences between concentrations obtained by the ATR and RID was -30 mg/dL, which approached zero, indicating no obvious bias between the ATR and RID methods. The 95 % confidence interval ranged from −670–611 mg/dL, which was relatively small in comparison with the range of IgG concentrations (~3000 mg/dL) obtained from the RID assay. Gauges of precision for the ATR and RID analytical methods are summarized graphically in Fig. 5a and b. The mean CV* for ATR analyses was 20 % and the mean CV* for RID analyses was 8.6 %. The RPD and RER values were estimated at 2.7 and 9.1, respectively.

### ATR sensitivity and specificity for detection of FTPI

The test characteristics of ATR assay were determined for diagnosis of FTPI (serum IgG <1000 mg/dL) in the prediction and entire data sets. The sensitivity, specificity, and accuracy are shown in Table 3. Within the entire data set, the number of samples that had IgG concentrations from the RID assay of <1000 mg/dL was 102 of 200 samples, resulting in a true FTPI prevalence of 51 %. The number of samples that had IgG concentrations <1000 mg/dL from ATR assay was 94 of 200 samples, resulting in an apparent FTPI prevalence of 47 %. There were no false positives and 8 false negatives identified (Table 3).

**Table 3:** Sensitivity, specificity, and accuracy of ATR-based IgG assay as a diagnostic test method to determine failure of transfer of passive immunity (FTPI) in the prediction (*n* = 67) and entire (*n* = 200) data sets

Data sets		Test characteristics						
	N	True positives	False positives	True negatives	False negatives	Se	Sp	Accuracy
Prediction	67	30	0	33	4	88 %	100 %	94 %
All data	200	94	0	98	8	92 %	100 %	96 %

*N* Number of samples, *Se* Sensitivity; *Sp* Specificity

## Discussion

In this study, the ATR assay was successfully developed as a tool for the rapid measurement of IgG concentrations in bovine serum. The analytical method development required the use of a large calibration data set with a wide range of IgG concentrations to develop a multivariate regression model that could be then applied to determine the IgG concentrations of new serum samples [42].

The performance of the analytical method depends on the spectral pre-processing approaches chosen [43]. To seek the optimal choice, different pre-processing strategies were evaluated and the most accurate was selected according to model performance metrics (i.e., lowest RMMCCV and *r* closet to 1) and confirmed by its high predictive accuracy (i.e., low RMSEP, high RPD and RER values) [41]. Regardless of the normalization method applied to PLS analysis, spectral smoothing was universally beneficial (Table 2). In contrast to other related studies [44], spectral derivation provided no improvement.

The ATR assay showed higher Pearson correlation and concordance coefficients than have been reported for previous transmission IR spectroscopy-based serum IgG assays for bovine [44], equine serum and plasma [10, 45], and alpaca serum [46]. Agreement between the ATR and RID assays was poorer at high IgG concentrations than at low IgG concentrations (Fig. 4). This may be attributed to the large number of serum samples with IgG concentrations below 1000 mg/dL (102 out of 200). As a result, the calibration model development was weighted towards low IgG concentrations, which are particularly more important for diagnosis of FTPI in farm animals [3, 4]. Similar findings have been observed for transmission IR spectroscopy-based serum IgG assays for bovine serum [44] and IR-based assays for other species [45, 46].

The precision of the ATR analytical method was found to be lower than that of the reference RID assay, as previously observed also for a transmission IR spectroscopy-based assay [44]. The relatively large CV* for the ATR assay typically occurs because the samples in the prediction set are not involved in the optimization of the calibration model (to ensure that the model performance is not overly optimistic). Nevertheless, given the conservative nature of this estimate of precision, the CV* of the IgG concentrations from the prediction samples lies within the acceptable range (should not exceed 20 %), according to the quality control standards of the US Food and Drug Administration Agency [47].

In anticipation of its application in the field, the ATR-based IgG assay was evaluated for its capacity to diagnose a clinically relevant problem - the occurrence of FTPI - using an IgG concentration cut-off value of 1000 mg/dL [3]. The ATR-based assay showed excellent sensitivity (0.92) and specificity (1.0), with values markedly better than those reported for a previously described transmission IR spectroscopy-based assay [44]. In comparison with other methods reported to assess FTPI in neonates, these results are equivalent to or better than most published assays [4, 13, 48]. The 8 false negatives for the ATR method corresponded to samples with RID-determined IgG values between 727–886 mg/dL, relatively close to the 1000 mg/dL diagnostic cut-off. These values indicate only partial FTPI, and thus the possible misdiagnosis of these animals poses a substantially lower risk of morbidity and mortality than would be the case for samples with lower IgG concentrations [48, 49]. There were no false positives identified by ATR spectroscopy. The very low false positive rate has previously been noted for transmission IR spectroscopy-based assays for camelids (no false positives out of 175 samples) [46], and bovine samples (4 false positives out of 200 samples) [44].

At present, the RID assay is acknowledged to be the reference standard test for quantification of IgG in bovine serum [8]. In practice, measurement of IgG by RID method is time consuming (18–24 h), utilizes reagents, and is expensive. In contrast, the ATR assay described in the current work is performed rapidly (one test can be completed within 3–4 min using 5 μL of sample) and the sample can be used with dilution in deionized water, the only required sample preparation step. These attractions, combined with practical advantages associated with compact, portable ATR spectrometers [16, 17], suggest the real possibility of using the technique in the field for assessing pre-calving assessment of dams, the management of colostrum, and ensuring adequate transfer of passive immunity to neonatal calves.

## Conclusions

Attenuated total reflectance infrared (ATR) spectroscopy in combination with multivariate data analysis is a feasible alternative for the rapid quantification of IgG concentrations in bovine serum and has the potential to effectively assess FTPI in neonatal calves. Testing of different pre-processing approaches revealed that spectral smoothing (without spectral derivation) significantly improved analytical performance and accuracy as compared to otherwise identical methods with no spectral smoothing

## Abbreviations

IgG: Immunoglobulin G
FTPI: Failure of transfer of passive immunity
ATR: Attenuated total reflectance
RID: Radial immunodiffusion
PLS: Partial least squares
RMMCCV: Root mean squared error Monte Carlo cross validation
RMSEC: Root mean squared error of calibration
RMSEP: Root mean squared error of prediction
CV: Coefficient of variation
RPD: Ratio of predictive deviation
RER: Range error ratio
